# Targeting Regulatory T Cells for Therapy of Lupus Nephritis

**DOI:** 10.3389/fphar.2021.806612

**Published:** 2022-01-06

**Authors:** Rajkumar Venkatadri, Vikram Sabapathy, Murat Dogan, Rahul Sharma

**Affiliations:** Center for Immunity, Inflammation and Regenerative Medicine (CIIR), Division of Nephrology, Department of Medicine, University of Virginia, Charlottesville, VA, United States

**Keywords:** IL-2, IL-6, IL-33, Treg cells, SLE, lupus nephritis

## Abstract

Lupus glomerulonephritis (LN) is a complex autoimmune disease characterized by circulating autoantibodies, immune-complex deposition, immune dysregulation and defects in regulatory T cell (Tregs). Treatment options rely on general immunosuppressants and steroids that have serious side effects. Approaches to target immune cells, such as B cells in particular, has had limited success and new approaches are being investigated. Defects in Tregs in the setting of autoimmunity is well known and Treg-replacement strategies are currently being explored. The aim of this minireview is to rekindle interest on Treg-targeting strategies. We discuss the existing evidences for Treg-enhancement strategies using key cytokines interleukin (IL)-2, IL-33 and IL-6 that have shown to provide remission in LN. We also discuss strategies for indirect Treg-modulation for protection from LN.

## Introduction

Systemic Lupus Erythematosus (SLE) is a debilitating autoimmune disease characterized by inflammation, increased circulating autoantibodies (autoAb), immune complex (IC) deposition, and multi-organ dysfunction affecting skin, joints, kidneys, brain etc. The cause of SLE is still unclear but genetic, environmental and hormonal factors have been linked to its predisposition. The prevalence of SLE is 366.6/100,000 in USA (2016 estimate) and 97/100,000 in UK (2012 estimate) (Barber et al., 2021). Lupus Foundation of America estimates that at least five million people worldwide may have some form of lupus (https://www.lupus.org/resources/lupus-facts-and-statistics). A majority of the patients affected by lupus progress to lupus nephritis (LN), an end stage renal disease marked by kidney IC deposition and glomerulosclerosis (reviewed in Ward, 2010). There are no approved medications that can cure the disease or provide long term remission with maintenance immunosuppression being the current option. There is thus an urgent need for novel treatment options for LN. Strategies for enhancing regulatory T cells (Treg) have garnered attention recently. While, many studies have employed Tregs for SLE, not enough work has specifically focused on lupus nephritis (LN). Therefore, we also included studies on Treg-based approaches for SLE.

## Autoimmunity and Tregs

**FIGURE 1:**
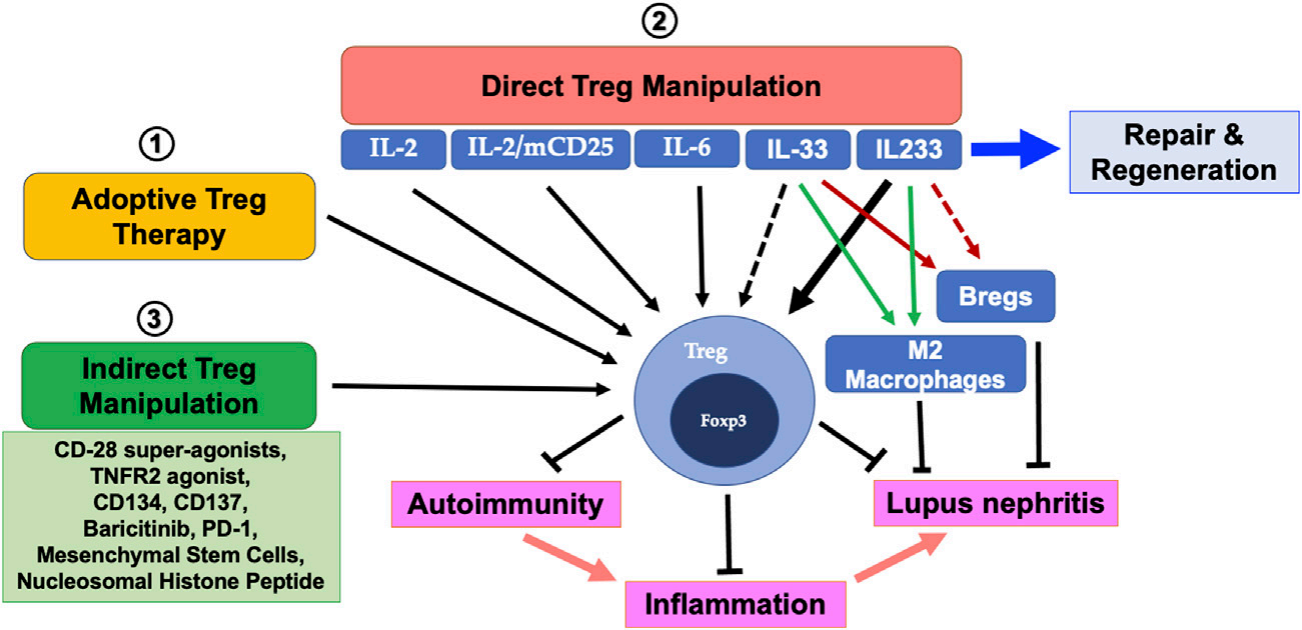
Treg supplementation therapies for autoimmunity and lupus nephritis (LN): **1)** Adoptive Treg transfer therapy has been successfully employed to attenuate inflammation and render protection in acute injury settings and recently, a similar approach was successfully employed in a SLE patient with beneficial outcomes. **2)** Cytokines IL-2, IL-2/mCD25, IL-6 and IL233 have been shown to directly cause robust expansion of Tregs which prevents autoimmunity and LN. Whether IL-33, which is reported to exhibit its protective effects on LN through modulation of M2 macrophages and Bregs have a direct effect on Tregs is yet to be elucidated. Similarly, IL233 has been shown to expand Tregs and induce a M2 phenotype but its effects on Bregs are currently unknown. **3)** Non-cytokine-based approaches using agonists, small molecules, mesenchymal stem cells and nucleosomal histone peptides have been shown to support Treg expansion to target LN.

Tregs constitute an important immune cell subset that prevents abnormal activation of the immune system and provide tolerance in allergy and autoimmunity (reviewed in Sharma and Kinsey, 2018). The role of Treg-deficiency in the onset and progression of autoimmunity has long been appreciated. In humans and mice, Tregs are identified as T-cells expressing high levels of IL-2 receptor alpha (CD25) and the transcription factor forkhead box P3 (Foxp3). Alongside IL-2 and CD25, which facilitate the development, function and stability of Tregs, numerous other cell-surface receptors for Tregs have been identified (reviewed in Sharma and Kinsey, 2018). IL-2 supports Treg development in the thymus (Vang et al., 2008) and is also required for their survival and function in the periphery (Fontenot et al., 2005; Barron et al., 2010; Sharma and Kinsey, 2018). While mechanisms of Treg-intrinsic function are still being recognized, factors including excessive production of pro-inflammatory cytokines and resistance to Treg-mediated suppression are being found to contribute to autoimmunity. A strong association of Treg defect in SLE and other human autoimmune diseases is now well-appreciated. Tregs not only control innate and adaptive immunity, but also regulate cellular damage and promote repair (reviewed in Shevach, 2018; Sharma, 2020). Thus, Treg-enhancement strategies remain an attractive strategy for remission from LN (Figure 1).

## Immune-Dysregulation in Lupus Nephritis

T cell activation is one of the major pathogenic mechanisms in the immune-dysregulation observed in lupus patients (reviewed in Mountz et al., 2019). Lupus patients have an elevated number of T effector cells, indicating a continued pathogenic response triggered either by pathogens that may mimic self-antigen (Zhao et al., 2019a; Múnera et al., 2020; Munroe et al., 2020), uncleared self-antigen from dying cells (reviewed in Muñoz et al., 2010; Prechl et al., 2016), or altered cytokine milieu contributing to effector cell bias (reviewed in Bengtsson and Rönnblom, 2017; Paquissi and Abensur, 2021). The role of complement proteins in LN is quite complex, where tissue damage is induced by complement deposition, and hypocomplementemia of C3 and C4 is pronounced in active disease, suggesting that complement-mediated clearance of autoantigen and IC play a central role in pathogenesis (reviewed in Li et al., 2021). The effector T-cells on one hand can induce cell-mediated organ dysfunction directly, they also activate innate-immune cells including macrophages and dendritic cells (DC) for fueling the immune dysregulation, as well as differentiate in to T-follicular helper (TfH) cells to help auto-reactive B-cells to produce high-affinity autoAb (reviewed in Mountz et al., 2019; Chen and Tsokos, 2021). The role of cytokines in lupus is supported by reports of elevated Tumor necrosis factor (TNF)-α and Interferon (IFN)-γ in SLE patients (Harigai et al., 2008). Other proinflammatory mediators including IL-6 and IL-17 lead to TH17/Treg dysregulation in lupus patients compared to healthy individuals (Tang et al., 2019).

## IL-2: A Master Regulator of Tregs in Lupus Nephritis

IL-2 was initially discovered as a growth factor that promoted T-cell proliferation *in vitro* (Morgan et al., 1976), but was later realized to be more critical for Treg-maintenance (Fontenot et al., 2005) and T-cell regulation (reviewed in Chen and Tsokos, 2021). IL-2 deficiency may contribute to autoimmunity in SLE patients and lupus-prone mice by inducing paucity of Tregs, defective activation-induced cell death (AICD) and increased IL-17 production (Lippe et al., 2012). Two back-to-back reports first showed the induction of IL-17 producing TH17 cells by TGFβ and IL-6 (Bettelli et al., 2006; Mangan et al., 2006). Subsequently, Laurence et al. showed that IL-2 through STAT5 directly inhibits the STAT3-mediated TH17 differentiation, because blocking of IL-2 or deletion of STAT5 in CD4 T-cells enhanced RORγT, a key transcription factor for TH17 differentiation (Yang et al., 2011). The role of IL-2/STAT5 in TH2 responses as well as inhibition of TH1 was first showed by W. E. Paul and colleagues (reviewed in Zhu et al., 2006). Accordingly, in the setting of multi-organ inflammatory disease in the Foxp3-mutant scurfy mice, it was shown that deletion of IL-4 or STAT6 (which is downstream to IL-4/IL-13 signaling) enhanced TH1 responses, thus showing the protective effects of TH2 in autoimmune inflammation in several organs (Sharma et al., 2011; Ju et al., 2012). Recently, Hsiung et al. (2020) showed that inflammation leads to lower levels of IL-2R signaling in Tregs, which further lowers their suppressive function. They concluded that low dose IL-2 therapy could possibly overcome the negative effects of inflammation on Tregs. Several investigators have tried to utilize the ability of IL-2 to expand Tregs for protection in autoimmune diseases (Fontenot et al., 2005; Bayer et al., 2007; Grinberg-Bleyer et al., 2010). However, in a study using the NZB/W mice, long-term treatment with a low dose of 1,500 units/day IL-2 or a high dose of 15,000 units/day did not alter the active lupus-like disease or splenocyte populations (Owen et al., 1989). In an interesting approach, a fusion protein of IL-2 with IL-2Ra (CD25) joined by a linker and termed mIL-2/CD25 inhibited LN in female NZB x NZW and male MRL/lpr mice (Xie et al., 2021). The treatment induced Treg expansion without elevation of NK, CD4^+^/CD8^+^ T cells and proinflammatory cytokines.

In clinical studies treatment of SLE patients with low dose IL-2 increased Tregs, decreased AICD and restored CTL responses along with a reduction of serum anti-dsDNA antibodies (von Spee-Mayer et al., 2016; He et al., 2016). Another clinical study in refractory SLE patients reported that the SLEDAI scores (a disease activity index for lupus patients) were significantly lowered with low dose IL-2 and rapamycin treatment and normalization of TH17/Treg ratios were significantly reduced (Zhao et al., 2019b). An interesting study focusing on the safety and efficacy of low dose IL-2 in 11 autoimmune disease in 46 patients including SLE found significant Treg-expansion without effector T-cell activation (Rosenzwajg et al., 2019). While low dose IL-2 has shown promise for SLE patients, several study results are still pending. Patients often receive multiple injections due to the short half-life of IL-2 and there is a concern of side effects triggered by low dose IL-2 mediated activation of cytotoxic lymphocytes and NK cells.

### Low Dose IL-2 Therapy for Other Autoimmune Diseases

IL-2 therapy has also been successfully tested in other lesser studied autoimmune diseases. A Phase I/II clinical trial on low dose IL-2 therapy in HCV-induced vasculitis increased Treg levels and reduced vasculitis (Saadoun et al., 2011). In a study on Alopecia areata (Castela et al., 2014), low dose IL-2 achieved local recruitment of Treg and also caused regrowth of scalp hair in the majority of participants. This study emphasized the importance of Treg supplementation in conditions involving skin lesions. In a study focusing on autoimmune hepatitis, low dose IL-2 normalized liver enzyme levels (Lim et al., 2018). The TRANSREG clinical trial that comprised of patients with 11 autoimmune diseases also showed promising findings with low dose IL-2 administration (Rosenzwajg et al., 2019). Other reports in primary Sjogren’s syndrome (Miao et al., 2018), Polymyositis/Dermatomyositis (Zhang et al., 2021), Psoriatic arthritis (Wang et al., 2020) and Amyotrophic lateral sclerosis (Camu et al., 2020) also showed benefits of low dose IL-2 therapy.

## IL-33: An Alarmin Cytokine That Regulates Innate Immunity and Inflammation

Interleukin-33 was identified as a nuclear cytokine belonging to the IL-1 family that is known to induce proinflammatory cytokines and expression of integrins on leukocytes and endothelial cells (Dinarello, 2009; Spooner et al., 2013). The pathway for IL-33 and its receptor ST2 is actively being pursued for immune modulation during autoimmune and inflammatory diseases (Monticelli et al., 2011; Byers et al., 2013). Two forms of ST2: transmembrane (mST2) and soluble isoforms (sST2) have been described (Mueller and Jaffe, 2015). The sST2 has been identified as a biomarker for cardiac, pulmonary and graft-versus-host diseases (Bajwa et al., 2015; Bayes-Genis et al., 2015; Xiao and Zheng, 2016). Recent studies have demonstrated a protective role of IL-33/ST2 in several inflammatory settings (Miller et al., 2008; Mato et al., 2009; Milovanovic et al., 2012). In the hTNF.Tg model of inflammation, IL-33-treatment induced alternatively activated macrophages (AAM) and inhibited TNFα-induced bone-loss (Zaiss et al., 2011). Interestingly, IVIG treatment was shown to suppress autoimmunity through IL-33 induced TH2 responses (Anthony et al., 2011), suggesting that IL-33/ST2 pathway may activate anti-inflammatory mechanisms in other settings. Prior studies on IL-33 have established that recombinant IL-33, both *in vitro* and *in vivo*, expanded ST2^+^ Tregs. *In vitro*, these ST2^+^ Tregs were more efficient in suppressing IL-12/IL-33 driven CD8^+^ T cell IFNγ production than their ST2^-^ counterpart (Matta et al., 2014). Early IL-33 administration in lupus prone NZB/W F1 mice was shown to induce regulatory B cells and reduce autoantibody levels. Additionally, an M2 macrophage signature was also induced implying a regulatory role of IL-33 in lupus onset (Mohd Jaya et al., 2020). Contrastingly, in a previously published study, antibody-mediated IL-33 neutralization suppressed disease in lupus prone mice along with an increase in Tregs and reduced IL-17 levels (Li et al., 2014). There is thus a disconnect in the available literature on the effects of IL-33 and its role in LN warrants further investigation.

## IL-6: A Paradoxical Target for Treg Expansion

IL-6 was originally identified as a B-cell differentiation factor that affects the autoantibody and cytokine production. IL-6 plays a key role in defense to infections by regulating the proinflammatory and regulatory T cells as well as contribute to autoimmunity. Elevated serum IL-6 levels correlate with disease severity in SLE patients (Chun et al., 2007). Pathological role of IL-6 has been demonstrated in disease development via targeting IL-6 with anti-IL-6 antibodies or by gene knockout approaches by several investigators. Indeed, IL-6 suppression was shown to rescue from rheumatoid arthritis (Narazaki et al., 2017), SLE (Tackey et al., 2004), scleroderma (O’Reilly et al., 2013) and many other diseases. Both TH17 and Tregs require TGFβ for their differentiation through induction of both RORγt and Foxp3 expression, where IL-6 negatively regulates Tregs by promoting TH17 (Zhou et al., 2008). The regulation of TH17/Treg differentiation by IL-6 explains the involvement of this pro-inflammatory cytokine in diseases that exhibit a prominent TH17 signature as a causative factor. While underlying mechanism for the observed efficacy of IL-6 blockade therapies are yet to be uncovered, the paradigm that IL-6 is associated with disease initiation and progression stands challenged by recent observations from Steinmetz and colleagues for a protective role of IL-6 in LN (Hagenstein et al., 2019). In 2016, Tregs dually expressing the transcription factors Foxp3 and RORγt were identified and were given the term “biTregs” for their roles in nephrotoxic nephritis (NTN) model of LN (Kluger et al., 2016). It was found that RORγt^+^Foxp3^+^ biTregs expanded with LN in the spleens and kidneys. The expansion of this subset of cells were higher than the expansion of conventional Tregs (cTregs: Foxp3^+^ Tregs) and IL-17^+^ Tregs. Further characterization also confirmed that the biTregs did not differentiate into cTregs or Th17 cells and that the IL-17 production by these cells was dependent on RORγt. It was concluded that the biTregs are a novel bifunctional Treg lineage with distinct properties and maybe a novel therapeutic target for LN. The observation that IL-6 did not inhibit Treg development or activation in the disease setting, but rather enhanced T effector activation that caused loss of Treg-mediated suppression led Steinmetz group to focus on the biTregs as an active effector Treg lineage (Nish et al., 2014; Ohnmacht et al., 2015; Sefik et al., 2015; Kluger et al., 2016; Yang et al., 2016). Utilizing IL-6 knockout mice, Hagenstein and colleagues (Hagenstein et al., 2019) further showed that IL-6 is required for the anti-inflammatory function of Tregs, as IL-6 treatment resulted in a significant expansion of RORγt^+^ biTregs and correlating with protection against LN.

## IL233: A Novel Hybrid Cytokine for LN Remission

IL-2 defect parallels with progression of autoimmunity and LN, and also correlates with reduced levels of Tregs. We found that IL-2 also regulates the expression of IL1RL1 (ST2; IL-33 receptor) on CD4 T-cells (Sharma et al., 2011). In addition, it was found that a major subset of Tregs expresses ST2 and that IL-33 expands Tregs *in vitro* and *in vivo*. Importantly, a novel cooperation between IL-2 and IL-33 was identified, which protected against inflammatory diseases by expanding, activating and mobilizing Tregs more efficiently than either cytokine alone. For more efficient restriction of this cooperativity to Tregs, a fusion cytokine of IL-2 and IL-33 (termed IL233) was generated (Stremska et al., 2017). IL233 exerted a sustained increase in Tregs, specifically in the renal lymph nodes and protected mice from LN more efficiently than IL-2 and IL-33 injected either alone or together. IL233 treatment not only prevented onset of LN, but also induced lifelong remission in moderate to severely proteinuric NZM2328 mice without any detectable side effects. IL233 treatment also inhibited the progression of LN in proteinuric MRL/lpr mice and protected them from early mortality (Stremska et al., 2019). The mechanisms, in addition to increasing Tregs, suggested restoring of IL-2 production by T-cells and tolerance induction via reduced expression of CD80 and CD86 on macrophages and DC. Interestingly, autoAb production and IC deposition were not significantly inhibited, suggesting a disconnect between autoAb and end-organ failure (Clynes et al., 1998; Ge et al., 2013). Thus, IL233 treatment demonstrated therapeutic potential in IFNα-accelerated and spontaneous LN in NZM2328 as well as MRL/lpr mice, indicating its general applicability.

## Alternate Strategies for Treg Expansion

A number of alternate strategies are reported for *in vivo* Treg activation and expansion. These included the use of Treg-related cell surface proteins, signaling and epigenetic modulation by small molecules, as well as approaches using auto-antigen peptides and microbes. CD28 is a co-stimulatory molecule expressed on T-cells and engaged by CD80/CD86 on antigen presenting cells for activation and cell cycle. In a rat model of anti-Thy1 nephritis, it was observed that CD28 super-agonists (CD28SA) at low levels efficiently expanded Tregs, and decreased proteinuria and serum creatinine levels (Miyasato et al., 2011). The roles of TNFRs (Tumor Necrosis Factor Receptors) are being recognized and TNFR2 is highly expressed in Tregs. TNF-α is a pleiotropic cytokine, which exists in both membrane-bound and soluble forms. The membrane bound TNF-α preferentially interacts with TNFR2 and results in suppressive function due to lack of cytoplasmic death domain. In a model of chronic inflammation, use of TNFR2 specific agonist TNCscTNF80 resulted in Treg expansion and reduced inflammation in mouse model of rheumatoid arthritis (Schmid et al., 2017). Similar to TNRF2, Ox40 (CD134) is expressed on Tregs and effector T cells. Ox-40 expression was elevated in several T-cell subsets in proteinuric NZB/W F1 mice. While, treatment with antagonistic Ox40 mAb accelerated autoAb and LN, administration of an agonistic Ox40:Fc fusion protein in an IFNα-accelerated lupus model significantly delayed the onset of severe proteinuria and improved the survival, suggesting a benefit of targeting this pathway (Sitrin et al., 2017). However, the effect of Ox40:Fc on Tregs was not evaluated, despite earlier observations that Ox40-deficient mice had reduced Tregs and that Ox40L overexpression increased Tregs in the spleen without disease suppression, probably due to evasion of Treg-mediated suppression by Ox40 engagement on effector T-cells (Takeda et al., 2004), Analogous to CD134, CD137 is highly expressed on Tregs and a study by Sun et al., showed that treatment with agonistic monoclonal antibody to CD137 blocked spontaneous autoimmunity in MRL/lpr mice (Sun et al., 2002), likely by promoting Treg expansion (Zhang et al., 2007).

Baricitnib, a selective inhibitor of Janus kinase (JAK1) and JAK2 is approved for use in rheumatoid arthritis. In a recent study, Lee et al. observed that treatment with baricitnib could effectively attenuate lupus-like phenotype in MRL/lpr mice. This was accompanied by reduction of total, CD8^+^ and abnormal double-negative T-cells along-with a greater proportion of follicular (CXCR5^+^) and extra-follicular (CXCR5^−^) Tregs, which could have afforded protection (Lee et al., 2021). Checkpoint inhibitors are currently being tested for treatment of several forms of cancer and their effects on Tregs remains controversial. Interestingly, in NZB/W F1 mice, anti-PD1 antibody treatment reduced CD4^+^PD-1^+^ T cells, promoted suppressive capability of Tregs and ameliorated LN (Wong et al., 2013). Mesenchymal stem cells (MSCs) are multipotent progenitor cells exerting immunosuppressive capacity with respect to both innate and adaptive immune response. MSCs are used in clinical settings to treat various lupus like conditions for over a decade (Zhou et al., 2020). Gazdic et al. (2018) demonstrated that indoleamine 2,3-dioxygenase (IDO) secreted from MSCs stimulates expansion and activation of Tregs to produce IL-10. Whether such effects can have therapeutic efficacy in LN setting is yet to be explored.

Several autoantigen peptide-based approaches are being developed to deliver these molecules to autoreactive T cells to promote clonal deletion or to develop immunoregulation. Kang et al. (2005) showed that a very low-dose of nucleosomal histone peptide efficiently controlled lupus in SNF1 lupus prone mice and induced Treg-subsets. The role of microbiome and probiotics in immune-regulation of SLE is also being actively researched. It was found that certain *Clostridium* strains reduced TH17 cells and induced Tregs. Further, *Bifidobacterium bifidum* supplementation prevented CD4 T-cell overactivation to restore Treg/Th17/Th1 imbalance in SLE via expanding Foxp3^+^IL-17^+^ populations, however, its effect on LN is still pending (López et al., 2016).

## Conclusions

As of today, there are 173 clinical trials ongoing for therapy of LN. The field of lupus research, having seen some promising therapeutics is now fraught with termination of ongoing trials due to unwarranted effects. There still is no approved drug that can be safely used long term to treat patients with LN. Tregs can be targeted effectively for LN remission, however, there are limited studies focusing on Treg supplementation and its probable adverse effects, if any. With the identification of key cytokines and alternative strategies to regulate Treg function, expansion and activation, it is imperative to continue research efforts on Treg-based strategies for remission from LN.
